# AI Dependence and Self-Reported Creativity Among Chinese College Students: The Roles of Motivation for AI Use and Perceived AI Literacy

**DOI:** 10.3390/bs16091712

**Published:** 2026-09-21

**Authors:** Jie Hou, Yanhong Huang, Qing Wang, Xiaoxue Yao, Peizheng Zhang, Fengqing Zhao

**Affiliations:** 1 School of Education and Cognitive Science, Zhengzhou University, Zhengzhou 450001, China; houj189@zzu.edu.cn (J.H.); huangyanhong@stu.zzu.edu.cn (Y.H.); uazpz@gs.zzu.edu.cn (P.Z.); 2 Psychological Counseling Center, Capital Normal University, Beijing 100048, China; 6589@cnu.edu.cn; 3 Faculty of Education, Zhengzhou Normal University, Zhengzhou 450044, China; xiaoxue@zznu.edu.cn

**Keywords:** AI dependence, self-reported creativity, motivation for AI use, perceived AI literacy, college students, moderated mediation

## Abstract

Creativity is a critical higher-order cognitive capacity for college students in the age of artificial intelligence. This study examined the relationship between AI dependence and self-reported creativity among Chinese college students, the mediating roles of motivation for AI use and the moderating role of AI literacy. A total of 5764 valid responses were collected from university students across multiple regions of China. Participants completed measures of AI dependence, perceived AI literacy, motivation for AI use, and self-reported creativity. The results showed that AI dependence was negatively associated with self-reported creativity. Parallel mediation analysis indicated significant negative indirect associations through escape and social motivation, whereas the indirect associations through instrumental and entertainment motivation were positive. In the primary moderated mediation analysis, perceived AI literacy significantly moderated the associations of AI dependence with escape, social, and entertainment motivation, but not with instrumental motivation. The interaction between AI dependence and perceived AI literacy in predicting self-reported creativity was not significant. The study contributes to research on AI use in higher education by identifying differentiated motivational pathways and clarifying the pathway-specific moderating role of perceived AI literacy.

## 1. Introduction

The rapid diffusion of artificial intelligence (AI), particularly general AI, has increasingly embedded AI tools in college students’ learning, academic work, and everyday problem solving. In higher education, this development raises an important question concerning creativity, a higher-order cognitive capacity involving the generation of original and useful ideas, the evaluation of alternatives, and the solution of ill-structured problems. This question is theoretically and practically important because general AI may both augment and displace the cognitive processes through which creative ideas are generated. Existing findings remain mixed. Some studies suggest that general AI can support idea generation, creative exploration, and divergent thinking (X. Wang et al., 2025; Doshi & Hauser, 2024; Lee & Chung, 2024), whereas others warn that general AI may foster cognitive offloading, cognitive miserliness, overreliance, or reduced diversity of ideas, thereby weakening creative thinking (Boers et al., 2025; Sternberg, 2024; H. Wang et al., 2024; Zhai et al., 2024). These inconsistent findings indicate that AI’s relationship with creativity is unlikely to be uniform or mechanically deterministic; rather, it may depend on students’ psychological motives, patterns of engagement, and individual capacities.

Most prior studies have focused on whether students use general AI or how frequently they use it (e.g., Doshi & Hauser, 2024; Ma et al., 2024; Qi et al., 2024; M. Zhou & Peng, 2025). This usage-centered perspective may overlook a more consequential shift: the movement from active, instrumental AI use to passive AI dependence. University students’ dependence on artificial intelligence has shown an increasing trend over time (Chen et al., 2025). Drawing on research on smartphone dependence and internet addiction, AI dependence can be understood as a pattern in which individuals habitually rely on AI to cope with pressure, avoid challenges, or replace higher-order cognitive activities that would otherwise require self-directed effort, such as deep thinking and creative ideation (Adegbesan et al., 2024; Zhai et al., 2024; X. Zhou et al., 2024). The present study therefore examines AI dependence as a key predictor of creativity among Chinese university students.

To explain how AI dependence may be associated with creativity, this study focuses on motivation for AI use and AI literacy. AI dependence is not itself a psychological mechanism; its cognitive and behavioral consequences are likely to be shaped by why students turn to AI. From the perspectives of Self-Determination Theory and Uses and Gratifications Theory, AI use may be driven by functional needs, social needs, emotional regulation, or avoidance-oriented motives (Huang et al., 2024). In addition, Students’ AI literacy may condition whether and how they recognize the affordances and risks associated with AI dependence (Zhai et al., 2024; Cui & Zhang, 2026). Accordingly, this study proposes a moderated parallel mediation model in which four types of motivation for AI use mediate the association between AI dependence and creativity, and AI literacy serves as a moderator.

### 1.1. AI Dependence and Creativity

Artificial intelligence dependence (hereafter referred to as “AI dependence”) refers to individuals’ excessive reliance on AI technologies and applications across multiple domains, including academic work, daily life, and social interaction, which is manifested not only in the frequent use of AI tools but also in a pronounced psychological attachment to them (H. Zhang et al., 2024). Existing studies have preliminarily examined the prevalence and developmental level of AI dependence among university students, as well as its negative consequences and influencing factors. A survey of 750 university students from five cities, including Shanghai, showed that more than 50% of students used conversational artificial intelligence (CAI) for over two hours per day, and more than 74.8% used CAI almost every day. These findings suggest that CAI has become deeply embedded in students’ daily learning and life, characterized by high-frequency and long-term use (Chen et al., 2025).

Unlike ordinary AI use, AI dependence implies a shift from deliberate tool use to reliance that may substitute for autonomous cognitive effort. For university students, this distinction is especially relevant because creativity requires sustained attention, divergent thinking, tolerance of uncertainty, and the ability to elaborate ideas independently (Liu & Zhang, 2024; Marrone et al., 2024; H. Wang et al., 2024). AI dependence may undermine creativity when students use AI as a default substitute for idea generation, problem representation, or reflective evaluation (Boers et al., 2025; Liu & Zhang, 2024; Sternberg, 2024). In such cases, students may conserve effort in the short term but weaken the cognitive processes through which originality and flexible thinking are developed. Thus, we formed the first hypothesis:

**Hypothesis** **1.** *AI dependence is negatively associated with creativity among university students.*

### 1.2. Motivation for AI Use as an Indirect Pathway

Based on empirical studies of AI motivation and classic psychological theories, Huang et al. (2024) classified motivations for AI use into four categories: escape motivation, social motivation, instrumental motivation, and entertainment motivation (Cho, 2019; Choi & Drumwright, 2021; Y. L. Ng & Lin, 2022). These categories provide a useful framework for examining the psychological pathways through which AI dependence may be associated with creativity.

Escape motivation refers to the use of AI to avoid real-life stress, negative emotions, or academic anxiety (Huang et al., 2024). According to Self-Escape Theory and Internet Compensation Theory, individuals who experience unmet psychological needs or excessive pressure may turn to technology for compensatory relief. When students use AI primarily to escape from difficulties, their engagement with challenging tasks may become more passive (S. Wang & Huang, 2024; Wan et al., 2025). Prior work suggests that avoidant technology use can reduce cognitive engagement in demanding creative tasks and inhibit the development of creative thinking (Roskes et al., 2012).

**Hypothesis** **2.** *There is a negative indirect association between AI dependence and self-reported creativity through escape motivation.*

Social motivation refers to the tendency to use AI to satisfy needs for interpersonal connection, belongingness, and social recognition (Huang et al., 2024). As reliance on AI increases, students may increasingly turn to AI not only for task-related assistance but also to satisfy social and emotional needs, making socially motivated use one possible psychological function associated with AI dependence. Research based on the compensation hypothesis suggests that interaction with AI may alleviate social anxiety and loneliness to some extent (Tang et al., 2023; Kim et al., 2025). Other scholars caution that excessive reliance on AI for social needs may replace some real interpersonal interaction and may impair social skill development over time (Pilato et al., 2008; Y. Zhang et al., 2025). In the university context, however, socially motivated AI use may also function as a low-pressure form of interaction or emotional support, thereby reducing anxiety and preserving psychological resources for creative cognition.

**Hypothesis** **3.** *There is a positive indirect association between AI dependence and self-reported creativity through social motivation.*

Instrumental motivation refers to the use of AI for functional and goal-oriented purposes, such as acquiring knowledge, improving efficiency, and solving problems (Huang et al., 2024). A central form of instrumental motivation is information seeking. In academic contexts, students who use generative AI for literature searching, data analysis, or hypothesis refinement may engage in deep processing when they critically integrate AI-generated content with their own knowledge structures (Benvenuti et al., 2023; Ma et al., 2024; Qi et al., 2024; Wan et al., 2025). Such reflective integration can support complex ideation and creative problem solving.

From a cognitive offloading perspective, individuals may rely on external tools to reduce the cognitive demands of a task, particularly when the internal cognitive demands required to perform the task are high (Risko & Gilbert, 2016). Moderate and critical AI use may reduce lower-level cognitive burden and free resources for higher-order thinking and creative elaboration (Lee & Chung, 2024; X. Wang et al., 2025; Zong et al., 2025). Thus, instrumental motivation may constitute an adaptive pathway linking AI dependence with creativity when students retain critical evaluation and active integration (Chen et al., 2025; Xu et al., 2025).

**Hypothesis** **4.** *There is a positive indirect association between AI dependence and self-reported creativity through instrumental motivation.*

Entertainment motivation refers to the use of AI to seek enjoyment, relaxation, emotional regulation, or leisure (Huang et al., 2024). According to Effort-Recovery Theory and Conservation of Resources Theory, individuals need psychological detachment after sustained cognitive demands in order to restore depleted psychological resources (Sonnentag & Fritz, 2007). AI-based entertainment may temporarily distance students from academic pressure, reduce psychological tension, and support emotional recovery; however, this pathway may be beneficial only when leisure use does not become avoidance or passive substitution (S. Wang & Huang, 2024; Zong et al., 2025). Through this recovery process, entertainment motivation may indirectly benefit creative thinking.

**Hypothesis** **5.** *There is a positive indirect association between AI dependence and self-reported creativity through entertainment motivation.*

### 1.3. AI Literacy as a Moderator

AI literacy may constitute an important boundary condition in the relationship between AI dependence and creativity. According to Long and Magerko’s (2020) AI literacy competency framework, AI literacy involves a broad set of competencies, including understanding the capabilities and limitations of AI systems, critically interpreting data, and making ethically informed judgments. Consistent with this view, AI literacy has been conceptualized as a multidimensional capacity that encompasses not only knowledge and use of AI systems but also critical evaluation, ethical awareness, and responsible decision-making (Long & Magerko, 2020; D. T. K. Ng et al., 2021).

Based on the Competence × Risk Exposure Model, digital competence can simultaneously expand opportunities and increase exposure to risks (Helsper, 2012; Livingstone & Helsper, 2013). Recent work on AI literacy and creativity suggests that literacy may operate through complex motivational and engagement pathways rather than exerting a simple protective effect (Benvenuti et al., 2023; M. Zhou & Peng, 2025). Empirical evidence supports this view. Students with higher AI literacy are generally better able to judge the value, suitability, limitations, and ethical implications of generative AI tools (Al-Abdullatif & Alsubaie, 2024; Kong et al., 2023; O’Dea et al., 2026). At the same time, over-reliance on AI dialogue systems may undermine critical thinking, independent analysis, and creativity when students accept AI-generated outputs uncritically (Zhai et al., 2024). Thus, AI literacy may not simply weaken the effects of AI dependence; rather, it may shape the pathways through which AI dependence is translated into motivational orientations and creative outcomes.

From a risk-sensitivity perspective, students with higher AI literacy may be more alert to the potential costs of AI dependence, including cognitive offloading, reduced cognitive autonomy, and diminished originality. As a result, AI literacy may attenuate dependence-driven escape, social, and entertainment motives, because students with higher literacy are more likely to recognize these motives as forms of avoidance, substitution, or superficial gratification. Conversely, AI literacy may strengthen instrumental motivation, because students with higher AI literacy are better able to use AI strategically for academic learning, idea development, and problem solving. In this sense, AI literacy may redirect AI-dependent engagement away from nonproductive gratification-oriented motives and toward more goal-directed instrumental use.

**Hypothesis** **6.** *AI literacy moderates the indirect association between AI dependence and creativity through motivation for AI use.*

AI literacy may also moderate the direct association between AI dependence and creativity. Although AI literacy may protect students by improving their critical awareness and regulatory capacity, students with both high AI dependence and high AI literacy may engage with AI tools more extensively and elaborately, thereby increasing their exposure to the cognitive costs of dependence. Intensive AI-assisted work may increase the need to evaluate, verify, and regulate AI-generated content, which may impose additional cognitive burden and reduce the cognitive autonomy required for original ideation. Therefore, after accounting for motivational pathways, AI literacy may intensify rather than buffer the negative direct association between AI dependence and creativity.

**Hypothesis** **7.** *AI literacy moderates the direct association between AI dependence and creativity.*

### 1.4. The Present Study

This study aims to examine the indirect pathways and boundary conditions characterizing the association between AI dependence and self-reported creativity among Chinese university students. First, it examines the overall association between AI dependence and creativity. Second, it tests the parallel mediating roles of four motivations for AI use: escape, social, instrumental, and entertainment motivation. Third, it examines whether AI literacy moderates the first-stage paths from AI dependence to the four motivational mediators and the direct path from AI dependence to creativity.

## 2. Materials and Methods

### 2.1. Participants and Procedure

The study targeted full-time undergraduate and graduate students enrolled in the fall semester of 2024. A convenience sampling strategy, supplemented by snowball recruitment, was employed. Questionnaires were distributed and organized through classmates, colleagues, and on-campus contacts of the research group at multiple universities, using both paper-based administration and online surveys. The sample covered 37 universities across various regions, including Beijing, Shanghai, Zhengzhou, Guiyang, and other cities. A total of 5916 questionnaires were initially collected. Cases with extreme values (|Z| > 4) on key study variables, including completion time, age, and scale scores, were excluded (*n* = 147). An additional five cases were excluded because of data-entry errors, resulting in a final analytic sample of 5764 participants (97.43% of the initial sample). Participants ranged in age from 16 to 38 years (M = 20.87, SD = 2.44). Demographic information was available for 5760 participants. Of these participants, 1668 were male (28.96%), 4078 were female (70.80%), and 14 did not report gender (0.24%). Regarding educational stage, 2259 were first- or second-year undergraduates (39.22%), 2315 were third- or fourth-year undergraduates (40.19%), and 1186 were master’s or doctoral students (20.59%). Regarding major, 1661 students majored in the humanities (28.84%), 1958 in the social sciences (33.99%), and 2141 in the natural sciences (37.17%). Regarding hometown background, 3386 reported an urban background (58.78%), 606 a township background (10.52%), 1762 a rural background (30.59%), and 6 did not report hometown background (0.10%).

The study protocol was approved by the institutional review board (IRB). All participants provided written informed consent prior to participation. The final sample included two 16-year-old university students who provided their own written informed consent. The IRB was subsequently consulted regarding these two participants and confirmed that their consent procedure was acceptable and that their data could be retained in the study. The survey was conducted independently of course grades or academic performance. All participants were informed prior to the survey that their participation was voluntary and that they could withdraw at any time without any adverse consequences.

### 2.2. Measures

#### 2.2.1. AI Dependence

AI dependence was measured using the Chinese version AI Dependence Scale adapted by Huang et al. (2024) from the revised Smartphone Addiction Scale. The scale consists of five items, including “I have tried to reduce the amount of time I spend on AI but failed” and “I feel anxious and upset when I cannot use artificial intelligence.” Each item was rated on a 4-point Likert scale ranging from 1 (strongly disagree) to 4 (strongly agree). Total scores were calculated, with higher scores indicating higher levels of AI dependence. In the present study, McDonald’s omega for the scale was 0.889. The model fit test showed that, χ^2^/df = 45.19, CFI = 0.99, TLI = 0.97, and SRMR = 0.01, all meeting the recommended criteria (>0.90/0.95). The RMSEA value of 0.09 was marginally acceptable. Taken together, the overall model fit generally met the psychometric requirements. The standardized factor loadings of all items ranged from 0.60 to 0.87, CR = 0.88, indicating good internal consistency reliability; and AVE = 0.59, exceeding the threshold of 0.50, suggesting that the latent variable possessed good convergent validity.

#### 2.2.2. Perceived AI Literacy

Perceived AI literacy was measured using the AI Literacy Scale (AILS) adapted from B. Wang et al. (2023), who developed and validated a 12-item instrument for assessing individual competence in using AI technology. The study was administered in Chinese. The original scale was in English and was translated by teachers with professional backgrounds in both psychology and English. The scale comprises four constructs: awareness (e.g., “I can distinguish between smart devices and non-smart devices”), use (e.g., “I can skillfully use AI applications or products to help me with my daily work”), evaluation (e.g., “I can evaluate the capabilities and limitations of an AI application or product after using it for a while”), and ethics (e.g., “I always comply with ethical principles when using AI applications or products”). Each item was rated on a 7-point Likert scale ranging from 1 (strongly disagree) to 7 (strongly agree). Confirmatory factor analysis (CFA) was performed to examine the factor structure of the scale. After deleting three items with factor loadings below 0.30, the model demonstrated acceptable fit: χ^2^/df = 31.38, CFI = 0.95, TLI = 0.91, RMSEA = 0.07, SRMR = 0.03. Factor loadings range from 0.67 to 0.90. The substantial correlation between four dimensions (*rs* = 0.68–0.95) indicates considerable shared variance among the four dimensions. Thus, the overall composite score was used to represent overall AI literacy, which is also supported by previous studies (Zhu et al., 2026; Yu et al., 2026). In the present study, McDonald’s omega for the scale was 0.936. The average variance extracted (AVE) and composite reliability (CR) for the four dimensions were as follows: Awareness (AVE = 0.63, CR = 0.77), Usage (AVE = 0.63, CR = 0.77), Evaluation (AVE = 0.73, CR = 0.89), and Ethics (AVE = 0.61, CR = 0.76).

#### 2.2.3. Motivation for AI Use

Motivation for AI use was measured using the Chinese version revised AI Use Motivation Scale developed by Huang et al. (2024). The scale comprises four dimensions. Escape motivation refers to using AI to avoid or retreat from real-life difficulties and stressors (e.g., “I use AI as a way to escape from family, friends, or other problems”). Social motivation reflects the desire to use AI to meet needs for companionship and social interaction (e.g., “I use AI as a way to avoid being alone”). Instrumental motivation involves using AI as a tool for practical goals, such as information seeking and knowledge acquisition (e.g., “I use AI to search for and obtain the information I need”). Entertainment motivation refers to using AI for enjoyment, relaxation, and leisure (e.g., “I use AI to entertain myself and relax”). Each dimension contains three items, rated on a 4-point Likert scale ranging from 1 (strongly disagree) to 4 (strongly agree). In the present study, McDonald’s omega for the overall scale was 0.879. The McDonald’s omega coefficients for escape, social, instrumental, and entertainment motivation were 0.929, 0.910, 0.851, and 0.932, respectively. Confirmatory factor analysis (CFA) supported the four-factor structure, with acceptable model fit: χ^2^/df = 39.83, CFI = 0.97, TLI = 0.96, RMSEA = 0.08, SRMR = 0.05. The standardized factor loadings ranged from 0.73 to 0.95, all reaching moderate-to-high levels. The average variance extracted (AVE) and composite reliability (CR) for the four factors were as follows: Escape (AVE = 0.81, CR = 0.93), Social (AVE = 0.72, CR = 0.89), Instrumental (AVE = 0.66, CR = 0.85), and Entertainment (AVE = 0.82, CR = 0.93).

#### 2.2.4. Self-Reported Creativity

Self-reported creativity was measured using the Creativity Scale for College Students validated and refined by He et al. (2015). The original scale was in Chinese. The scale includes three factors: divergent thinking, intellectual application ability, and personality traits. It contains 17 items, such as “I like to ask questions that others have not thought of.” Each item was rated on a 5-point Likert scale ranging from 1 (never) to 5 (always). In the present study, McDonald’s omega for the scale was 0.920. Confirmatory factor analysis (CFA) was conducted to evaluate the factor structure of the second-order structure of the creativity scale. The model demonstrated the overall reasonableness of the measurement model, χ^2^/df = 22.74, CFI = 0.91, TLI = 0.90, RMSEA = 0.06, SRMR = 0.05. The three standardized second-order factor loadings were 0.90, 0.89, and 0.65. The factor loadings of first-order items range from 0.47 to 0.84. The average variance extracted (AVE) and composite reliability (CR) for the three dimensions were as follows: Divergent Thinking (AVE = 0.51, CR = 0.87), Intelligence (AVE = 0.56, CR = 0.89), and Personality Traits (AVE = 0.44, CR = 0.69). Given that our substantive analyses relied on the total creativity score rather than on individual dimension scores, this localized AVE issue does not materially affect the interpretation of the main findings.

### 2.3. Data Analysis

First, missing values in cases with less than 20% missing data were handled using multiple imputation (MI) in IBM SPSS Statistics (version 21.0; IBM Corp., Armonk, NY, USA). Ten imputed datasets were generated using fully conditional specification. The imputation model included AI dependence, the four motivational dimensions, perceived AI literacy, and self-reported creativity. Parameter estimates and standard errors were pooled across the imputed datasets using Rubin’s rules.

To test the hypothesized moderated parallel mediation model, we first used the lavaan package (version 0.6-21) in R (version 4.0.0) to estimate and compare the indirect effects through four specific mediation paths: AI dependence → escape motivation → self-reported creativity, AI dependence → social motivation → self-reported creativity, AI dependence → instrumental motivation → self-reported creativity, and AI dependence → entertainment motivation → self-reported creativity. We then used Model 8 of the PROCESS macro for SPSS (version 4.0; Hayes, 2022) to examine whether AI literacy moderated the first-stage paths from AI dependence to the four motivational mediators and the direct path from AI dependence to self-reported creativity. AI dependence was specified as the independent variable (X), self-reported creativity as the dependent variable (Y), escape motivation, social motivation, instrumental motivation, and entertainment motivation as parallel mediators (M1–M4), and AI literacy as the moderator (W). Prior to the moderated mediation analysis, all continuous variables were z-standardized (M = 0, SD = 1). The interaction terms were subsequently constructed from the standardized variables. The significance of direct, indirect, and conditional indirect effects was assessed using bootstrap procedures with 5000 resamples. Effects were considered statistically significant when their 95% bias-corrected confidence intervals did not include zero. The VIF values for AI dependence, escape motivation, social motivation, instrumental motivation, entertainment motivation, and AI literacy are 1.71, 3.25, 3.05, 1.31, 1.34, and 1.31, indicating that multicollinearity was not a concern in the present study.

To assess the robustness of our findings to random sample composition, we performed a subsampling stability analysis by randomly excluding 7.4% of observations in 1000 iterations (matching the actual proportion of paper-and-pencil responses in our original data, 440/5916) and re-estimated all models. The core coefficients remained stable across all iterations in both magnitude (all coefficients within ±5%) and statistical significance (all *p* < 0.001).

## 3. Results

### 3.1. Common Method Bias

Common method bias was assessed using Harman’s single-factor test (H. Zhou & Long, 2004). Exploratory factor analysis identified nine factors with eigenvalues greater than 1. The first factor accounted for 24.35% of the variance, below the recommended 40% threshold. Therefore, common method bias was not considered a serious concern in this study. To examine the potential threat of common method bias, we conducted a confirmatory factor analysis (CFA) comparing the theoretical 12-factor model with a single-factor model. The theoretical 12-factor model demonstrated a good fit to the data (χ^2^/df = 19.77, CFI = 0.92, TLI = 0.90, RMSEA = 0.06, SRMR = 0.05). In contrast, the single-factor model yielded a poor fit (χ^2^/df = 144.03, CFI = 0.30, TLI = 0.26, RMSEA = 0.16, SRMR = 0.20), which was substantially worse than that of the theoretical model. These results suggest that common method bias does not pose a significant concern in this study.

### 3.2. Descriptive Statistics and Correlation Analysis

Overall, AI dependence, escape motivation, social motivation, and entertainment motivation were significantly below the scale midpoint (all *p*s < 0.001, Cohen’s *d*s ranging from −1.760 to −0.213). In contrast, instrumental motivation was significantly above the scale midpoint (*p* < 0.001, Cohen’s *d* = 0.341, 95% CI [0.315, 0.368]), and self-reported creativity (*p* < 0.001, Cohen’s *d* = 1.022, 95% CI [0.990, 1.053]) was significantly above the scale midpoint.

The self-reported creativity scores were approximately normally distributed (skewness = 0.211, kurtosis = 0.283), with only 2.8% of respondents at the maximum score and 54% at or above 3.5. These values suggest that ceiling compression is unlikely to be a serious issue in our data.

Bivariate correlation analyses showed that AI dependence, escape motivation, social motivation, instrumental motivation, and entertainment motivation were positively correlated with one another. AI literacy and self-reported creativity were negatively correlated with AI dependence, escape motivation, and social motivation, but positively correlated with instrumental motivation and entertainment motivation. Detailed results are presented in Table 1. Considering the high correlation between escape motivation and social motivation, discriminant validity was assessed using the improved HTMT_2_ criterion proposed by Roemer et al. (2021). The results showed that the HTMT_2_ value between escape motivation and social motivation was 0.866, indicating good discriminant validity between the two constructs.

To assess potential clustering of students within universities, unconditional models were estimated for all key study variables. The ICCs ranged from approximately 0 to 0.019 (see Supplementary Materials Table S1), indicating minimal between-university variation. Given the negligible clustering, single-level regression models were retained for the primary analyses.

### 3.3. Preliminary Analysis: Parallel Multiple Mediation

An observed-variable parallel multiple mediation model was estimated using the lavaan package (version 0.6-21) in R with maximum likelihood estimation and 5000 bootstrap resamples. Scale or subscale composite scores were entered as observed variables in the model. The total effect of AI dependence on self-reported creativity was significant and negative (β = −0.165, Boot SE = 0.013, 95% Boot CI [−0.195, −0.143], *p* < 0.001). After the four mediators were controlled, the direct effect remained significant and negative (β = −0.122, Boot SE = 0.010, 95% Boot CI [−0.155, −0.089], *p* < 0.001). Thus, the association between AI dependence and self-reported creativity was partially explained by the mediating variables, while a residual direct effect remained.

The specific indirect effects, presented in Table 2, showed divergent mediating pathways. The indirect effects through escape motivation and social motivation were significant and negative (β = −0.075, 95% Boot CI [−0.103, −0.048], β = −0.022, 95% Boot CI [−0.046, −0.0006]). The indirect effects through instrumental motivation and entertainment motivation were significant and positive (β = 0.012, 95% Boot CI [0.007, 0.018]; β = 0.038, 95% Boot CI [0.031, 0.046], respectively).

### 3.4. Primary Analysis: Moderated Parallel Multiple Mediation

The moderated mediation model was tested using PROCESS Model 8 with 5000 bootstrap samples. The analysis examined whether AI literacy moderated the first-stage paths from AI dependence to the four mediators and/or the direct path from AI dependence to self-reported creativity. The path regression results are shown in Table 3. The model explained 21.1% of the variance in self-reported creativity, R^2^ = 0.211, F(7, 5756) = 220.427, *p* < 0.001.

For the direct association between AI dependence and self-reported creativity, the interaction of AI dependence and perceived AI literacy was not significant (b = −0.006, SE = 0.007, *t* = −0.906, *p* = 0.365, 95% CI [−0.019, 0.007]).

**Figure 1:**
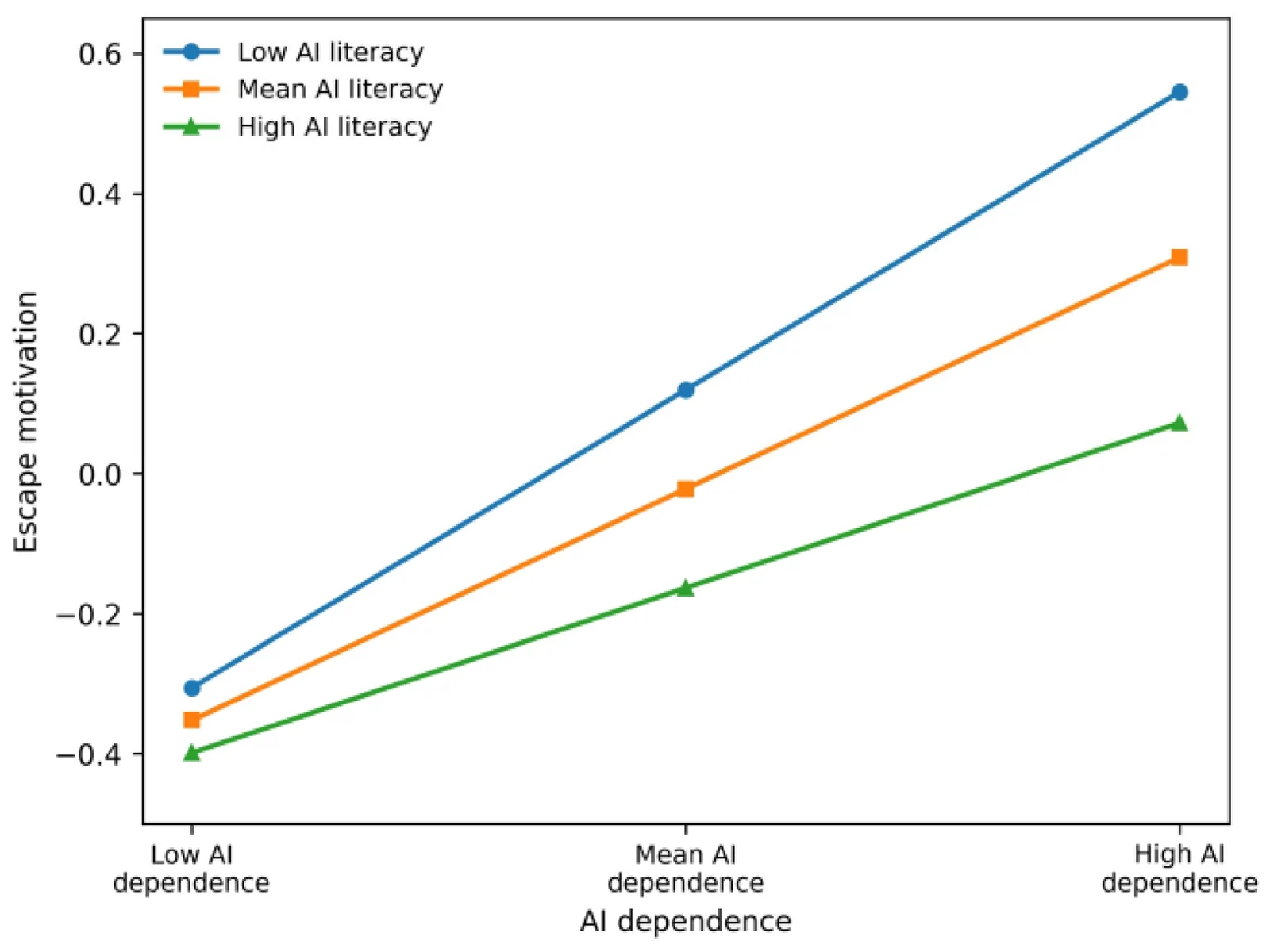
Interaction between AI dependence and AI literacy on escape motivation.

**Figure 2:**
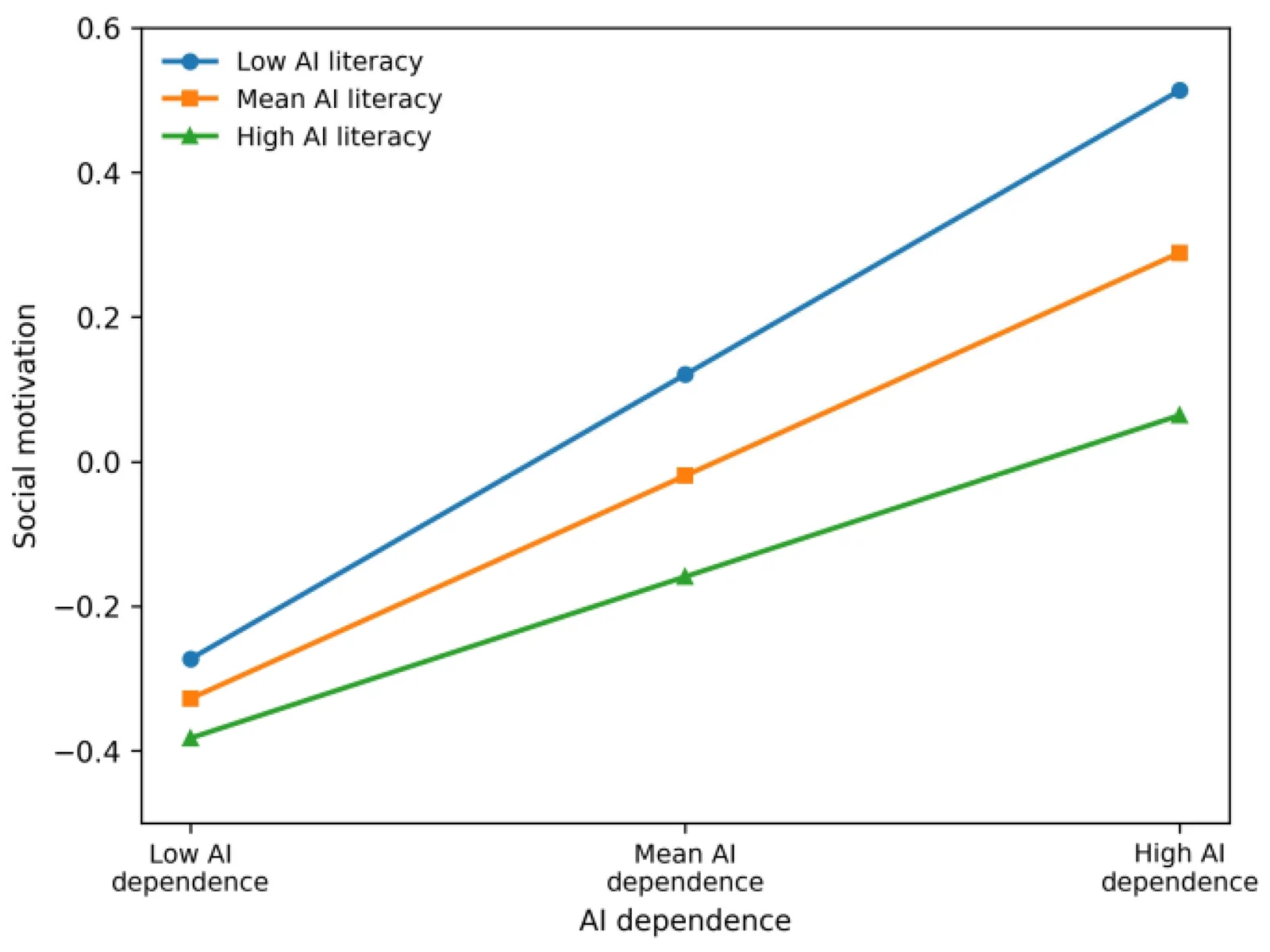
Interaction between AI dependence and AI literacy on social motivation.

**Figure 3:**
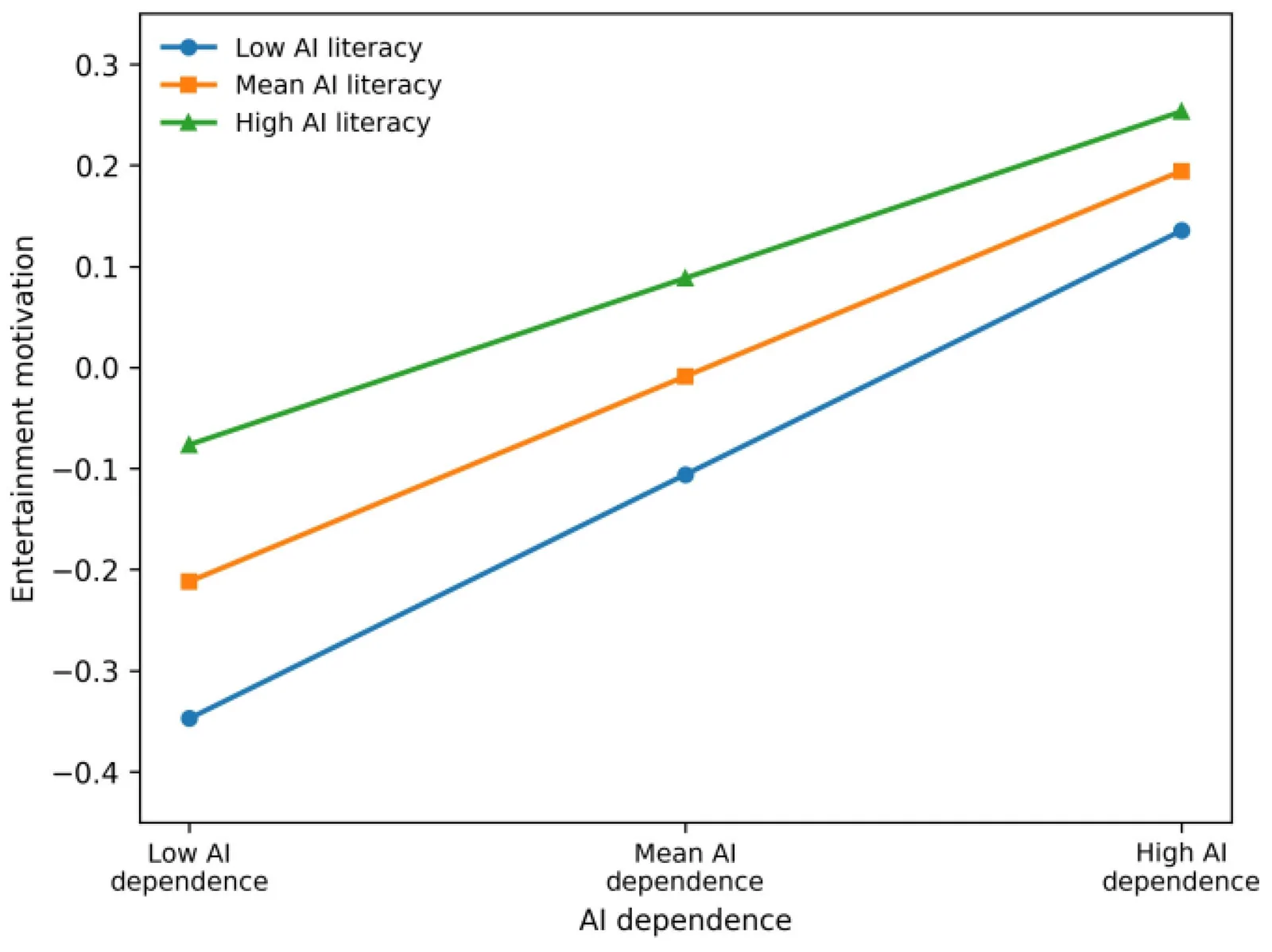
Interaction between AI dependence and AI literacy on entertainment motivation.

For the first-stage paths, AI literacy significantly moderated the association between AI dependence and three of the four motivational mediators. The interaction terms between AI dependence and AI literacy were significant for escape motivation (b = −0.163, *p* < 0.001), social motivation (b = −0.135, *p* < 0.001), and entertainment motivation (b = −0.045, *p* < 0.001), but not for instrumental motivation (b = 0.014, *p* = 0.228). The significant interaction patterns are shown in Figure 1, Figure 2 and Figure 3. The interaction plots display predicted values at low (−1 SD), mean, and high (+1 SD) levels of perceived AI literacy.

For the second-stage paths, escape motivation (b = −0.032, *p* = 0.010), social motivation (b = 0.026, *p* = 0.030), instrumental motivation (b = −0.028, *p* < 0.001), and entertainment motivation (b = 0.084, *p* < 0.001) were all significantly associated with self-reported creativity.

The conditional indirect effects at different levels of AI literacy are presented in Table 4. For the path through escape motivation, the index of moderated mediation was significant and positive (index = 0.005, BootSE = 0.002, 95% Boot CI [0.001, 0.009]). The negative indirect effect remained significant at low (−1 SD; effect = −0.0232, 95% Boot CI [−0.0412, −0.0052]), mean (effect = −0.0180, 95% Boot CI [−0.0321, −0.0040]), and high (+1 SD; effect = −0.0129, 95% Boot CI [−0.0233, −0.0028]) levels of perceived AI literacy. Pairwise contrasts indicated that the indirect effect was stronger at low AI literacy than at mean AI literacy (contrast = 0.005, 95% Boot CI [0.001, 0.009]) and in turn than high AI literacy (contrast = 0.005, 95% Boot CI [0.002, 0.019]), indicating that the moderated mediation became progressively weaker as perceived AI literacy increased.

For the path through social motivation, the index of moderated mediation was significant and negative (index = −0.004, BootSE = 0.002, 95% Boot CI [−0.007, −0.0003]), indicating that the indirect effect varied significantly as a function of perceived AI literacy. The positive indirect effect was significant at low (effect = 0.0162, 95% Boot CI [0.0013, 0.0313]), mean (effect = 0.0127, 95% Boot CI [0.0010, 0.0245]), and high (effect = 0.0092, 95% Boot CI [0.0007, 0.0179]) levels of perceived AI literacy. The pairwise contrasts among these conditional indirect effects were all significant, showing that the moderated mediation decreased as perceived AI literacy increased.

For the path through instrumental motivation, the index of moderated mediation was not significant (index = −0.0004, BootSE = 0.0004, 95% Boot CI [−0.0014, 0.0004]). The indirect effect was negative and significant at low (effect = −0.0062, 95% Boot CI [−0.0103, −0.0024]), mean (effect = −0.0066, 95% Boot CI [−0.0108, −0.0026]), and high (effect = −0.0070, 95% Boot CI [−0.0118, −0.0027]) levels of perceived AI literacy. Pairwise contrasts were not statistically significant.

For the path through entertainment motivation, the index of moderated mediation was significant and negative (index = −0.004, Boot SE = 0.001, 95% Boot CI [−0.006, −0.001]). The positive indirect effect was significant at low (effect = 0.0239, 95% Boot CI [0.0186, 0.0297]), mean (effect = 0.0201, 95% Boot CI [0.0157, 0.0250]), and high (effect = 0.0164, 95% Boot CI [0.0117, 0.0215]) levels of perceived AI literacy, and decreased as perceived AI literacy increased.

Furthermore, to examine the robustness of our findings against demographic confounds, we additionally ran a separate set of regression models that included sex, grade, hometown and major as covariates. The results show that the inclusion of these covariates did not alter the direction, magnitude, or statistical significance of any of the core predictors (see Supplementary Materials Table S2).

## 4. Discussion

This study examined the relationship between AI dependence and self-reported creativity among Chinese college students by integrating four motivations for AI use and AI literacy into a moderated parallel mediation model. Overall, AI dependence was negatively associated with self-reported creativity, with distinct indirect associations observed through different motivations for AI use. Perceived AI literacy further moderated some of the associations between AI dependence and these motivations. Taken together, the findings indicate that the association between AI dependence and self-reported creativity varies across motivations for AI use and, for some pathways, across levels of perceived AI literacy.

### 4.1. AI Dependence and Self-Reported Creativity

The first important finding is the significant negative association between AI dependence and self-reported creativity, which supported Hypothesis 1. This finding suggests that distinguishing AI dependence from AI use per se may be important when considering students’ self-reported creativity. Frequent or intensive AI use can be functional and goal-directed, whereas the dependence measure used in the present study captures psychological reliance, excessive use, withdrawal-related discomfort, and difficulty controlling AI use rather than use frequency alone. One possible explanation for the observed negative association is that reliance on AI may, in some contexts, reduce opportunities for independent idea generation, argument construction, or evaluative judgment. Such reduced cognitive engagement could potentially be associated with lower self-perceptions of creativity. However, these cognitive processes were not directly assessed in the present study. This interpretation is broadly consistent with previous arguments that overreliance on AI may be associated with reduced cognitive autonomy and shallower engagement (Boers et al., 2025; Liu & Zhang, 2024; Sternberg, 2024).

The present finding also provides a complementary perspective on mixed evidence from prior research. Experimental studies have shown that AI use can improve the quality or fluency of creative outputs in specific tasks (Doshi & Hauser, 2024; Lee & Chung, 2024; X. Wang et al., 2025). Such task-level improvements, however, address a different aspect of creativity from the self-reported tendencies assessed in the present study. Improved AI-assisted performance on a specific task does not necessarily imply stronger self-perceived creativity or broader creative capacity. The present findings therefore underscore the importance of distinguishing AI-supported creative performance, individuals’ self-perceptions of creativity, and creativity as a broader psychological construct.

### 4.2. The Indirect Association Through Escape Motivation

Escape motivation showed a negative indirect association between AI dependence and self-reported creativity, thus supporting Hypothesis 2. Students with higher AI dependence reported stronger escape motivation, and greater escape motivation was associated with lower self-reported creativity. This pattern is consistent with prior longitudinal evidence linking escape motivation with psychological distress and AI dependence (Huang et al., 2024) and suggests that the psychological function served by AI use may be relevant beyond the frequency or intensity of use alone.

One possible interpretation is that escape-oriented AI use may reflect an avoidance-related response to academic difficulty, uncertainty, or negative affect. Creative work often requires sustained engagement with ambiguity, unsuccessful attempts, and idea revision; using AI primarily to disengage from such discomfort may therefore be associated with fewer opportunities for sustained cognitive engagement. Related research has similarly linked shortcut-oriented or default acceptance of AI-generated content with reduced deep processing and lower research creativity (Wan et al., 2025). However, avoidance behavior, emotion regulation, and depth of cognitive processing were not directly assessed in the present study. These processes should therefore be regarded as possible explanations for the observed indirect association rather than mechanisms demonstrated by our data.

From an educational perspective, the findings suggest that attention to the motivational function of AI use may complement AI literacy education. Rather than focusing solely on how frequently students use AI, educators could incorporate metacognitive activities that help students recognize when they turn to AI primarily to avoid anxiety, uncertainty, or task difficulty. Potential strategies include reflective prompts, process-based assessment, and AI-use logs to help students monitor the purpose of their AI use and maintain active engagement with cognitively demanding tasks.

### 4.3. The Indirect Association Through Social Motivation

In the preliminary unconditional parallel mediation model, social motivation showed a small negative indirect association between AI dependence and self-reported creativity. In the moderated mediation model, however, the conditional indirect association through social motivation was positive and significant at low, mean, and high levels of perceived AI literacy, although its magnitude decreased as perceived AI literacy increased. Thus, H3 was not supported in the preliminary unconditional mediation model but received conditional support in the moderated mediation model. Given the small magnitude and model-dependent direction of this indirect association, the findings for social motivation should be interpreted cautiously.

One possible explanation for this pattern is that socially motivated AI use may serve heterogeneous functions. AI interaction could sometimes substitute for interpersonal exchange, potentially limiting access to social feedback and diverse perspectives that may contribute to creative thinking. Conversely, AI may provide a relatively low-pressure conversational setting for preliminary idea expression or feedback seeking before interaction with others. Previous research has suggested that human–AI interaction and interpersonal exchange may make different contributions to creative processes (Chen et al., 2025) and that generative AI can provide opportunities for idea exploration and articulation (Lee & Chung, 2024; H. Zhang et al., 2024). However, the present measure did not distinguish companionship-oriented, substitution-oriented, or collaboration-oriented forms of socially motivated AI use. These possibilities should therefore be regarded as tentative explanations rather than mechanisms demonstrated by the present findings.

The significant moderated mediation further indicates that the magnitude of the positive indirect association through social motivation varied across levels of perceived AI literacy, becoming weaker as perceived AI literacy increased. One possibility is that students with different levels of perceived AI literacy engage with socially motivated AI use differently; however, the present study did not directly assess how students interpreted, evaluated, or integrated AI-based social interactions. Future research should therefore distinguish different forms and functions of socially motivated AI use and directly examine how they interact with specific dimensions of AI literacy in relation to self-reported creativity.

### 4.4. The Indirect Association Through Instrumental Motivation

Hypothesis 4 was not supported. Although instrumental motivation was hypothesized to show a positive indirect association between AI dependence and self-reported creativity, the primary moderated mediation model showed a small negative indirect association at low, mean, and high levels of perceived AI literacy. The index of moderated mediation was not significant, indicating that the magnitude of this indirect association did not vary significantly as a function of perceived AI literacy. Thus, the observed pattern was contrary to our original expectation that instrumental motivation would constitute a positive pathway.

The findings for instrumental motivation also differed across model specifications: the preliminary unconditional mediation model yielded a small positive indirect association, whereas the partial association between instrumental motivation and self-reported creativity was negative after perceived AI literacy, the other motivational variables, and the theoretically specified interaction terms were included in the primary model. This sign reversal should therefore be interpreted cautiously and may reflect differences in model specification and shared variance among the predictors rather than a substantive change in the psychological function of instrumental AI use.

Beyond these model-specification considerations, one possible substantive interpretation is that instrumental AI use may vary in the degree of cognitive engagement it involves. Instrumental AI use may facilitate information access, knowledge integration, and idea generation, but reliance on readily available AI-generated information may also be associated with reduced cognitive elaboration when users accept outputs without further evaluation or transformation (Xu et al., 2025). Experimental and educational research similarly suggests that AI can support creative performance when it is used to explore alternatives, integrate information, and refine ideas (Lee & Chung, 2024; Benvenuti et al., 2023), whereas more shortcut-oriented use may be associated with shallower processing or limited transformation of AI-generated ideas (Wan et al., 2025; Boers et al., 2025).

### 4.5. The Indirect Association Through Entertainment Motivation

Entertainment motivation showed a positive indirect association between AI dependence and self-reported creativity, supporting Hypothesis 5. One possible interpretation is that entertainment-oriented AI use may provide opportunities for relaxation or temporary psychological detachment from academic demands. Recovery experiences have been associated with the restoration of affective and cognitive resources (Sonnentag & Fritz, 2007), while positive affect has been linked to greater cognitive flexibility and divergent thinking (Fredrickson, 2001; Baas et al., 2008). A second possible interpretation is that some forms of entertainment-oriented AI use may involve low-stakes exploration or playful engagement. Such playful engagement has been associated with creativity through greater exploration and unconventional combinations of ideas (Mainemelis & Ronson, 2006), and generative AI may provide opportunities to explore alternative ideas or modes of expression (Lee & Chung, 2024). However, the present study did not directly assess recovery experiences, positive affect, or playful exploration; these processes should therefore be regarded as tentative explanations rather than mechanisms demonstrated by the present findings. Notably, the magnitude of this positive indirect association decreased as perceived AI literacy increased, a pattern considered further in Section 4.6.

### 4.6. The Moderating Role of AI Literacy

In the primary moderated mediation model, perceived AI literacy significantly moderated the first-stage associations of AI dependence with escape, social, and entertainment motivation, but not with instrumental motivation. Consistent with these first-stage interactions, the indices of moderated mediation were significant for the indirect pathways through escape, social, and entertainment motivation, but not for the pathway through instrumental motivation. Thus, Hypothesis 6 received partial support. Specifically, the positive associations between AI dependence and escape, social, and entertainment motivation became weaker as perceived AI literacy increased. At the indirect-effect level, higher perceived AI literacy was associated with an attenuation of the negative indirect association through escape motivation and of the positive indirect associations through social and entertainment motivation. By contrast, the interaction between AI dependence and perceived AI literacy in predicting self-reported creativity was not significant. Thus, Hypothesis 7 was not supported, and the present findings do not indicate that perceived AI literacy significantly moderates the direct association between AI dependence and self-reported creativity.

One possible interpretation of the significant first-stage interactions is that students with different levels of perceived AI literacy may differ in how AI dependence is associated with particular motivations for continued AI use. The construct of AI literacy encompasses awareness of AI systems, competence in their use, critical evaluation of their capabilities and limitations, and ethical understanding (Long & Magerko, 2020; D. T. K. Ng et al., 2021). In the present study, these competencies were assessed through students’ self-reported perceptions of their AI literacy. Previous research has suggested that AI literacy may support more informed evaluation of AI-generated information and greater awareness of the opportunities and limitations of AI technologies (Al-Abdullatif & Alsubaie, 2024; Kong et al., 2023). Students’ perceptions of these competencies may be relevant to the weaker associations observed between AI dependence and some motivational dimensions at higher levels of perceived AI literacy. However, the present study did not directly assess metacognitive regulation, recognition of dependence-related risks, or regulation of emotion-driven AI use. These processes therefore remain possible explanations rather than mechanisms demonstrated by the present findings.

Importantly, the moderating effects were not uniform across motivational pathways, and their incremental explanatory contributions were generally small. Perceived AI literacy did not significantly moderate the association between AI dependence and instrumental motivation or the direct association between AI dependence and self-reported creativity. Accordingly, the findings should not be interpreted as evidence that higher perceived AI literacy necessarily buffers the negative correlates of AI dependence. Rather, they suggest that perceived AI literacy is associated with specific differences in some dependence–motivation relationships, the practical significance and underlying processes of which require further investigation.

### 4.7. Theoretical Contributions and Practical Implications

This study contributes to the literature in three ways. First, it shifts the focus from AI use frequency to AI dependence. This distinction is important because frequent AI use can be active, strategic, and reflective, whereas AI dependence implies a psychologically different pattern of reliance. Second, the study identifies differentiated motivational pathways. Escape motivation functioned as a maladaptive pathway, entertainment motivation functioned as a potentially restorative pathway, social motivation showed a conditional pattern, and instrumental motivation was more ambivalent than expected. Third, the study highlights the pathway-specific nature of perceived AI literacy’s moderating role, with generally small effect sizes. given the self-report nature of the AI literacy measure, these findings should be interpreted as preliminary evidence of differential associations rather than evidence of protective or risk-amplifying functions.

Several practical applications follow, though these should be viewed as proposals warranting future empirical evaluation. First, universities could consider teaching students to distinguish AI assistance from AI substitution. For instance, students might be encouraged to generate initial ideas independently before consulting AI and document how AI input was evaluated—but the effectiveness of such practices requires systematic investigation. Second, AI literacy programs could explore incorporating metacognitive activities such as reflective prompts or AI-use logs to help students identify the purpose of their AI use; however, these strategies would benefit from empirical evaluation. Third, educators might consider designing assignments that require process evidence, such as idea logs and reflection notes. Future research is needed to assess whether such designs effectively promote reflective engagement.

### 4.8. Limitations and Future Directions

Several limitations should be acknowledged. First, the cross-sectional design prevents causal conclusions. Although the proposed model was theoretically specified with AI dependence preceding motivations for AI use, alternative temporal orderings are also plausible. In particular, motivations for AI use may contribute to the development of AI dependence (Huang et al., 2024), and the associations among these constructs may also be reciprocal over time. Accordingly, the indirect associations identified in the present study should not be interpreted as evidence of a temporal or psychological mechanism. Future longitudinal studies with repeated assessments of AI dependence, motivations for AI use, and creativity, as well as experimental research where appropriate, are needed to compare alternative temporal models and clarify the directionality of these associations.

Second, the study relied on self-report measures. Though the measurement analyses provide evidence against severe common method bias, the cross-sectional and self-report nature of the data precludes definitive causal inference and may have inflated the observed associations to some extent. Especially, creativity was assessed using a self-report measure that captures students’ perceptions of their divergent thinking, intellectual application ability, and creativity-related personality characteristics. It therefore should not be interpreted as a direct measure of behavioral creative performance or the originality of actual products. Although self-report measures provide useful information about perceived creative tendencies, future research should combine them with performance-based assessments, such as divergent-thinking tasks or independent evaluations of creative products.

Third, although the AI dependence measure assessed difficulty regulating AI use and psychological reliance rather than use frequency, its adaptation from a smartphone addiction scale may not fully distinguish maladaptive dependence from intensive but functional AI engagement in academic contexts. Future research should combine dependence measures with behavioral indicators of AI use frequency, duration, and task context to better differentiate functional engagement from maladaptive reliance. Fourth, perceived AI literacy was self-reported and may reflect perceived rather than objective competence. The moderation effects were modest in magnitude (incremental R^2^ = 0.0002–0.028). Despite statistical significance for three pathways, the small effect sizes warrant cautious interpretation. Future research should employ objective literacy assessments and experimental designs. The educational strategies in Section 4.7 also require systematic testing before implementation.

In addition, because individual-level administration-mode identifiers were not retained in the anonymized analytic dataset, we could not empirically verify the missing-completely-at-random assumption underlying the subsampling analysis, and potential differences between paper-and-pencil and online administration remain a possible unmeasured confound.

## 5. Conclusions

This study found that AI dependence was negatively associated with self-reported creativity among Chinese college students and that motivations for AI use were associated with distinct indirect patterns. These patterns were further moderated by perceived AI literacy for escape, social, and entertainment motivation pathways. In addition, perceived AI literacy did not moderate the direct association between AI dependence and self-reported creativity. Given the cross-sectional and self-report design, the findings cannot be evidence of causal psychological mechanisms. Overall, the findings highlight the importance of distinguishing AI dependence from AI use per se and of considering both motivations for AI use and perceived AI literacy when examining students’ AI dependence and self-reported creativity.

## Figures and Tables

**Table 1:** Descriptive statistics and correlations among variables.

Variables	M	SD	1	2	3	4	5	6	7
1. AI dependence	1.78	0.57	1						
2. Escape motivation	1.47	0.58	0.62 ***	1					
3. Social motivation	1.54	0.63	0.54 ***	0.79 ***	1				
4. Instrumental motivation	2.77	0.80	0.15 ***	0.04 **	0.11 ***	1			
5. Entertainment motivation	2.32	0.85	0.21 ***	0.23 ***	0.37 ***	0.37 ***	1		
6. AI literacy	5.15	0.86	−0.23 ***	−0.35 ***	−0.32 ***	0.30 ***	0.07 ***	1	
7. Self-reported creativity	3.59	0.58	−0.17 ***	−0.18 ***	−0.13 ***	0.12 ***	0.14 ***	0.43 ***	1

Note. ** *p* < 0.01, *** *p* < 0.001.

**Table 2:** Results of the parallel multiple mediation analysis.

Path (Mediator)	Indirect Effect (β)	SE	95% Boot CI
Escape motivation	−0.075	0.014	[−0.103, −0.048]
Social motivation	−0.022	0.012	[−0.046, −0.0006]
Instrumental motivation	0.012	0.003	[0.007, 0.018]
Entertainment motivation	0.038	0.004	[0.031, 0.046]
**Pairwise contrasts of indirect effects**			
Escape motivation vs. social motivation	−0.054	0.024	[−0.100, −0.006]
Instrumental motivation vs. entertainment motivation	−0.026	0.005	[−0.037, −0.016]
Escape motivation vs. entertainment motivation	−0.114	0.015	[−0.143, −0.085]
Escape motivation vs. Instrumental motivation	−0.087	0.014	[−0.116, −0.060]
Social motivation vs. Instrumental motivation	−0.034	0.012	[−0.062, −0.013]
Social motivation vs. Entertainment motivation	−0.061	0.014	[−0.091, −0.037]

Note. Bold indicates the subsection heading “Pairwise contrasts of indirect effects”; all coefficients presented in the tables are standardized.

**Table 3:** Regression Analysis Results for the Moderated Mediation Models.

Outcome Variable	b	SE	t	*p*	95% CI	ΔR^2^	F Change
**Escape motivation**	R^2^ = 0.461, F(3, 5760) = 1644.763, *p* < 0.001						
AI dependence	0.567	0.010	57.053	<0.001	[0.548, 0.587]		
AI literacy	−0.243	0.010	−24.128	<0.001	[−0.263, −0.223]		
Int1	−0.163	0.009	−17.395	<0.001	[−0.181, −0.144]	0.028 ***	302.593
**Social motivation**	R^2^ = 0.353, F(3, 5760) = 1045.263, *p* < 0.001						
AI dependence	0.491	0.011	45.066	<0.001	[0.470, 0.513]		
AI literacy	−0.223	0.011	−20.201	<0.001	[−0.244, −0.201]		
Int2	−0.135	0.010	−13.191	<0.001	[−0.156, −0.115]	0.020 ***	174.013
**Instrumental motivation**	R^2^ = 0.140, F(3, 5760) = 311.933, *p* < 0.001						
AI dependence	0.235	0.013	18.735	<0.001	[0.211, 0.260]		
AI literacy	0.352	0.013	27.683	<0.001	[0.327, 0.377]		
Int3	0.014	0.012	1.207	0.228	[−0.009, 0.038]	0.0002	1.456
**Entertainment motivation**	R^2^ = 0.062, F(3, 5760) = 126.392, *p* < 0.001						
AI dependence	0.239	0.013	18.246	<0.001	[0.214, 0.265]		
AI literacy	0.115	0.013	8.632	<0.001	[0.089, 0.141]		
Int4	−0.045	0.012	−3.647	<0.001	[−0.069, −0.021]	0.002 ***	13.300
**Self-reported creativity**	R^2^ = 0.211, F(7, 5756) = 220.427, *p* < 0.001						
AI dependence	−0.049	0.009	−5.442	<0.001	[−0.066, −0.031]		
Escape motivation	−0.032	0.012	−2.562	0.010	[−0.056, −0.008]		
Social motivation	0.026	0.012	2.173	0.030	[0.003, 0.049]		
Instrumental motivation	−0.028	0.008	−3.603	<0.001	[−0.043, −0.013]		
Entertainment motivation	0.084	0.008	10.698	<0.001	[0.069, 0.110]		
AI literacy	0.239	0.008	30.009	<0.001	[0.223, 0.255]		
Int5	−0.006	0.007	−0.906	0.365	[−0.019, 0.007]	0.0001	0.821

Note. Bold indicates the outcome variable; Int = AI dependence × AI literacy interaction; *** *p* < 0.001.

**Table 4:** Conditional indirect effects of AI dependence on self-reported creativity at different levels of AI literacy.

Mediator	Level of AI Literacy	Indirect Effect	Boot SE	95% Boot CI
Escape motivation	Low	−0.023	0.009	[−0.041, −0.005]
	Mean	−0.018	0.007	[−0.032, −0.004]
	High	−0.013	0.005	[−0.023, −0.003]
	**Index**	0.005	0.002	[0.001, 0.009]
Social motivation	Low	0.016	0.007	[0.002, 0.031]
	Mean	0.013	0.006	[0.001, 0.025]
	High	0.009	0.004	[0.001, 0.018]
	**Index**	−0.004	0.002	[−0.007, −0.0004]
Instrumental motivation	Low	−0.006	0.002	[−0.010, −0.002]
	Mean	−0.007	0.002	[−0.011, −0.002]
	High	−0.007	0.002	[−0.012, −0.003]
	**Index**	−0.0004	0.0004	[−0.001, 0.0004]
Entertainment motivation	Low	0.024	0.003	[0.019, 0.030]
	Mean	0.020	0.002	[0.016, 0.025]
	High	0.016	0.002	[0.012, 0.021]
	**Index**	−0.004	0.001	[−0.006, −0.002]

Note. Bold indicates the index of moderated mediation. Low and high represent 1 SD below and 1 SD above the mean of perceived AI literacy, respectively.

## Data Availability

The data presented in this study are available on request from the corresponding author. The data are not publicly available due to ethical restrictions.

## Supplementary Materials

The following supporting information can be downloaded at: https://www.mdpi.com/article/10.3390/bs16091712/s1, Table S1: Variance Components and Intraclass Correlation Coefficients (ICCs) for Key Study Variables; Table S2: Hierarchical Regression Analysis Results for the Moderated Mediation Models.
