# Supplementary material for: AI Dependence and Self-Reported Creativity Among Chinese College Students: The Roles of Motivation for AI Use and Perceived AI Literacy
Source: Behav Sci (Basel). 2026 Sep 21;16(9):1712. doi: 10.3390/bs16091712 (PMC13603993; doi:10.3390/bs16091712)
Supplement: Supplementary file 1 [file behavsci-16-01712-s001.zip › behavsci-4439208-supplementary.pdf]

Supplementary Materials

Table S1. Variance Components and Intraclass Correlation Coefficients (ICCs) for Key Study Variables.

**Variable**	**τ^2（Between-school）**	**σ^2（Within-student）**	**ICC**
AI dependence	≈0	0.320	≈0
Escape motivation	≈0	0.340	≈0
Social motivation	≈0	0.394	≈0
Instrumental motivation	≈0	0.642	≈0
Entertainment motivation	≈0	0.720	≈0
AI literacy	≈0	0.737	≈0
Self-reported creativity	0.007	0.338	0.019

Table S2. Hierarchical Regression Analysis Results for the Moderated Mediation Models.

Outcome Variable	b	SE	t	*p*	95% CI	ΔR²	F change
**Escape motivation**			$R^2 = 0.461$, $F(9, 5738) = 547.756$, $p < 0.001$				
AI dependence	0.566	0.010	56.113	<0.001	[0.546, 0.586]		
AI literacy	−0.239	0.010	−23.338	<0.001	[−0.259, −0.219]		
Int1	−0.163	0.009	−17.332	<0.001	[−0.181, −0.114]		
Sex	−0.047	0.022	−2.096	0.036	[−0.091, −0.003]		
Lower-division undergraduates	−0.019	0.027	−0.724	0.469	[−0.071, 0.033]		
Upper-division undergraduates	−0.016	0.027	−0.612	0.541	[−0.068, 0.036]		
Hometown	0.030	0.020	1.460	0.145	[−0.010, 0.069]		
Humanities	0.047	0.025	1.891	0.059	[−0.002, −0.10]		
Social Sciences	0.064	0.024	2.668	0.008	[0.017, 0.111]	0.028 ***	300.384
**Social motivation**			$R^2 = 0.353$, $F(9, 5738) = 353.798$, $p < 0.001$				
AI dependence	0.491	0.011	45.066	<0.001	[0.470, 0.513]		
AI literacy	−0.223	0.011	−20.201	<0.001	[−0.244, −0.201]		
Int2	−0.135	0.010	−13.191	<0.001	[−0.156, −0.115]		
Sex	−0.039	0.025	−1.590		[−0.087, 0.009]		
Lower-division undergraduates	0.036	0.029	1.228	0.219	[−0.021, 0.093]		
Upper-division undergraduates	−0.068	0.029	−2.341	0.019	[−0.125, −0.011]		
Hometown	0.080	0.022	3.615	0.0003	[0.037, 0.123]		
Humanities	0.036	0.027	1.312	0.190	[−0.018, 0.090]		
Social Sciences	0.060	0.026	2.289	0.022	[0.009, 0.111]	0.019 ***	172.242
**Instrumental motivation**			$R^2 = 0.150$, $F(9, 5738) = 112.325$, $p < 0.001$				
AI dependence	0.235	0.013	18.735	<0.001	[0.211, 0.260]		
AI literacy	0.352	0.013	27.683	<0.001	[0.327, 0.377]		
Int3	0.014	0.012	1.207	0.228	[−0.009, 0.038]		
Sex	0.028	0.028	1.002		[−0.027, 0.084]		
Lower-division undergraduates	0.055	0.033	1.650	0.099	[−0.010, 0.121]		
Upper-division undergraduates	−0.039	0.033	−1.170	0.242	[−0.104, 0.026]		
Hometown	0.136	0.025	5.339	<0.001	[0.086, 0.186]		
Humanities	−0.116	0.032	−3.671	0.0002	[−0.178, −0.054]		
Social Sciences	−0.050	0.030	−1.665	0.096	[−0.109, 0.009]	0.0002	1.261
**Entertainment motivation**			$R^2 = 0.068$, $F(9, 5738) = 46.616$, $p < 0.001$				
AI dependence	0.239	0.013	18.246	<0.001	[0.214, 0.265]		
AI literacy	0.115	0.013	8.632	<0.001	[0.089, 0.141]		
Int4	−0.045	0.012	−3.647	<0.001	[−0.069, −0.021]		
Sex	0.026	0.030	0.884		[−0.032, 0.084]		
Lower-division undergraduates	−0.059	0.035	1.692	0.091	[−0.009, −0.128]		

Upper-division undergraduates	−0.105	0.035	−2.997	0.003	[−0.173, −0.036]		
Hometown	0.044	0.027	1.637	0.102	[−0.008, −0.096]		
Humanities	0.009	0.033	0.273	0.785	[−0.056, 0.074]		
Social Sciences	0.047	0.032	1.492	0.136	[−0.015, 0.109]	0.002 ***	13.041
**Self-reported creativity**			R^2 = 0.223, F(13, 5734) = 126.482, p < 0.001				
AI dependence	−0.049	0.009	−5.442	<0.001	[−0.066, −0.031]		
Escape motivation	−0.032	0.012	−2.562	0.010	[−0.056, −0.008]		
Social motivation	0.026	0.012	2.173	0.030	[0.003, 0.049]		
Instrumental motivation	−0.028	0.008	−3.603	<0.001	[−0.043, −0.013]		
Entertainment motivation	0.084	0.008	10.698	<0.001	[0.069, 0.110]		
AI literacy	0.239	0.008	30.009	<0.001	[0.223, 0.255]		
Int5	−0.006	0.007	−0.906	0.365	[−0.019, 0.007]		
Sex	−0.009	0.016	−0.625		[−0.040, 0.021]		
Lower-division undergraduates	−0.014	0.019	−0.747	0.455	[−0.050, 0.023]		
Upper-division undergraduates	−0.020	0.019	0.261	0.261	[−0.057, 0.016]		
Hometown	−0.089	0.014	−6.311	<0.001	[−0.117, −0.061]		
Humanities	0.080	0.018	4.562	<0.001	[0.046, 0.115]		
Social Sciences	0.082	0.017	4.861	<0.001	[0.049, 0.114]	0.0001	1.041

Note. Bold indicates the outcome variable. Grade level was dummy-coded with graduate students as the reference group (Lower-division undergraduates = 1, Upper-division undergraduates = 1, Graduate students = 0). Major was dummy-coded with Natural Sciences as the reference group (Humanities = 1, Social Sciences = 1, Natural Sciences = 0). CI = confidence interval; ΔR^2 = change in R^2. Sex was coded as 0 = male, 1 = female; Hometown was coded as 0 = urban, 1 = rural/township. The degrees of freedom for the F statistics are based on the effective sample size after listwise deletion (N = 5748) due to missing values in the covariates. The total sample size was 5764.
